# Large-scale preparation of active caspase-3 in *E. coli *by designing its thrombin-activatable precursors

**DOI:** 10.1186/1472-6750-8-92

**Published:** 2008-12-11

**Authors:** Hyo Jin Kang, Young-mi Lee, Yu-Jin Jeong, Kyoungsook Park, Mi Jang, Sung Goo Park, Kwang-Hee Bae, Moonil Kim, Sang J Chung

**Affiliations:** 1 Nanobiotechnology Division, University of Science and Technology (UST), Yuseong, Daejeon, 305-806, Korea; 2 BioNanotechnology Research Center, KRIBB, Yuseong, Daejeon, 305-806, Korea; 3 Translational Research Center, KRIBB, Yuseong, Daejeon, 305-806, Korea

## Abstract

**Background:**

Caspase-3, a principal apoptotic effector that cleaves the majority of cellular substrates, is an important medicinal target for the treatment of cancers and neurodegenerative diseases. Large amounts of the protein are required for drug discovery research. However, previous efforts to express the full-length caspase-3 gene in *E. coli *have been unsuccessful.

**Results:**

Overproducers of thrombin-activatable full-length caspase-3 precursors were prepared by engineering the auto-activation sites of caspase-3 precursor into a sequence susceptible to thrombin hydrolysis. The engineered precursors were highly expressed as soluble proteins in *E. coli *and easily purified by affinity chromatography, to levels of 10–15 mg from 1 L of *E. coli *culture, and readily activated by thrombin digestion. Kinetic evaluation disclosed that thrombin digestion enhanced catalytic activity (*k*_cat_/*K*_*M*_) of the precursor proteins by two orders of magnitude.

**Conclusion:**

A novel method for a large-scale preparation of active caspase-3 was developed by a strategic engineering to lack auto-activation during expression with amino acid sequences susceptible to thrombin, facilitating high-level expression in *E. coli*. The precursor protein was easily purified and activated through specific cleavage at the engineered sites by thrombin, generating active caspase-3 in high yields.

## Background

Multicellular organisms maintain homeostasis through a balance between cell proliferation and death. Apoptosis is a controlled cell death process crucial in a wide range of biological activities, such as normal cell turnover, immune system, embryonic development, metamorphosis, and chemical-dependent cell death [1]. Neuronal death due to aberrant apoptosis underlies the symptoms of various neurological disorders, such as Alzheimer's, Parkinson's and Huntington's diseases, stroke, amyotropic lateral sclerosis (ALS), multiple sclerosis (MS) and spinal muscular atrophy [2]. On the other hand, inactivation of apoptosis by blocking upstream death signals or inhibition of caspase activity by IAP complex formation is central to cancer development and cellular resistance of cells against anticancer agents [3-5].

Caspases, a family of cysteine proteases, play crucial roles in apoptosis, pro-inflammatory cytokine activation, and presumably, keratinocyte differentiation [6,7]. Following the initial identification of caspase-1 in 1992 by two different groups [8,9], eleven caspases in humans and 25 in other eukaryotes have been characterized over the last decade [10]. In mammals, caspases are translated as inactive zymogens. While caspases-8 and -10 are activated by death receptor-mediated signals (extrinsic apoptosis pathway), caspase-9 activity is stimulated by intracellular death signals, including cytochrome *c *released from mitochondria (intrinsic apoptosis pathway). Activated caspases subsequently convert procaspase-3 and -7 to fully active enzymes by specific proteolytic cleavage. Caspase-6 is activated after caspase-3. The three former caspases are known as apoptotic initiators, whereas the latter three are known as apoptotic effectors or executioners. Caspase-3 (also designated CED-3, murine ICE, and a protease resembling ICE/CPP32 in humans) is the first reported apoptotic effector, and cleaves the majority of cellular substrates in apoptotic cells [11-14]. Caspase-7 is very similar to caspase-3 in terms of structure and substrate specificity [6]. As caspase-3 and -7 are the final executioners of apoptosis, both inhibition and activation of catalytic activities are of significant interest as therapeutic strategies for neurodegenerative diseases and cancers [5,15-18].

Drug discovery research, including screening chemical libraries as well as structural and kinetic analyses, requires large-scale caspase-3 preparation. When expressed in *E. coli*, full-length caspase-3 (procaspase-3) undergoes presumable autoprocessing to yield the appropriate subunits characteristic of the active enzyme with only a marginal expression level, probably due to its cytotoxicity [19]. While full-length caspase-3 has been expressed in *Pichia pastoris *[20], the process is long and the yield is inadequate, compared to conventional protein expression method in *E. coli*. The most frequently employed large-scale caspase-3 preparation method includes separate expression of the two insoluble domains in *E. coli *and subsequent refolding of the two combined domains for the active enzyme [21]. This method showed significantly improved protein yield. However, such a costly and time-consuming refolding process is unsuitable for efficient large-scale production for drug discovery research. Here we describe a novel method for high-level expression and purification of caspase-3 precursors. The precursor was strategically engineered to lack auto-activation during expression with amino acid sequences susceptible to thrombin, facilitating high-level expression in *E. coli*. The precursor protein was activated through specific cleavage at the engineered sites by thrombin, generating active caspase-3. Furthermore, this protein efficiently digested endogenous caspase-3 substrate.

## Results and discussion

### Design of thrombin-activatable caspase-3 precursors for high-level expression in *E. coli*

**Figure 1:**
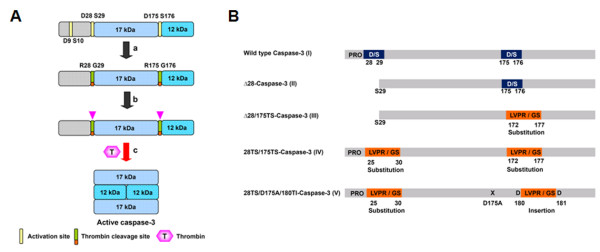
**High-level expression of caspase-3 precursors in *E. coli***. A. Design of thrombin-activatable caspase-3 precursors resistant to autoactivation. B. Structure of designed proteins. I: wild-type caspase-3; II: caspase-3 devoid of the N-terminal 28 amino acids; III: Six amino acids (172–177) of construct II were substituted with LVPRGS (a sequence susceptible to thrombin activity); IV: Two sites (amino acid sequences 25–30 and 172–177) of wild-type caspase-3 were replaced with LVPRGS. V: Six amino acids (172–177) of wild-type caspase-3 were mutated to LVPRGS, and an additional LVPRGS motif inserted between Asp180 and Asp181. Asp175 was mutated to Ala.

Caspases are translated in cells as inactive precursors, which are sequentially activated following internal or external cell death signals. Apoptotic initiators, such as caspases-8, -9 and -10, activated procaspases-3 and -7 by specific hydrolysis, thereby triggering cell death by specific apoptotic executioners [1,6,7]. During activation, procaspase-3 cleavage occurs at three sites, specifically, the C-terminal peptide bonds of Asp-9, Asp-28 and Asp-175 (Fig. 1A). As stated above, full activation of caspase-3 during overexpression in *E. coli *hampers large-scale preparation with conventional methods [19]. We planned to substitute the cleavage sites with specific peptide sequences that were resistant to caspase but susceptible to thrombin (Fig. 1A). Consequently, the precursor protein is not activated during expression, but stimulated by thrombin at a convenient time. The mutations designed included replacement of cleavage sites with LVPRGS, a well-known thrombin substrate sequence, and insertion of this sequence near cleavage sites with appropriate mutations of the cleavage site. Since procaspase-3 releases the N-terminal 28 amino acids during the activation, the matured caspase-3 (active caspase-3) does not have these 28 amino acids in its structure. Therefore, the constructs that lack the N-terminal 28 amino acids were prepared. Δ28-caspase-3 (**II**) was depleted of 28 amino acids at the N-terminus of procaspase-3 (**I**), while Δ28/175TS-caspase-3 (**III**) contained LVPRGS instead of wild-type residues at positions 172–177 of construct **II **(Fig. 1B). In the 28TS/175TS-caspase-3 construct (**IV**), LVPRGS mutations were introduced at two sites (positions 25–30 and 172–177) of wild-type caspase-3 (**I**). In 28TS/180TI-caspase (**V**), the LVPRGS motif replaced residues 25–30, and was inserted between Asp180 and Asp181, along with a D175A mutation to prevent auto-cleavage. Since caspase-3 activation occurs mainly via cleavage at Asp 28 and 175, constructs were designed to ensure cleavage by thrombin at the corresponding sites (between R and G of LVPRGS). The LVPRGS motif was inserted between Asp180 and 181 in 28TS/180TI-caspase (**V**) to maintain the structural integrity of the sequence encompassing residues 165–174 after cleavage, in view of the crystal structure of active caspase-3, which discloses tight interactions between this region and the other dimer within a heterotetrameric arrangement [22].

### Expression and purification of the engineered caspase-3 precursors

**Figure 2:**
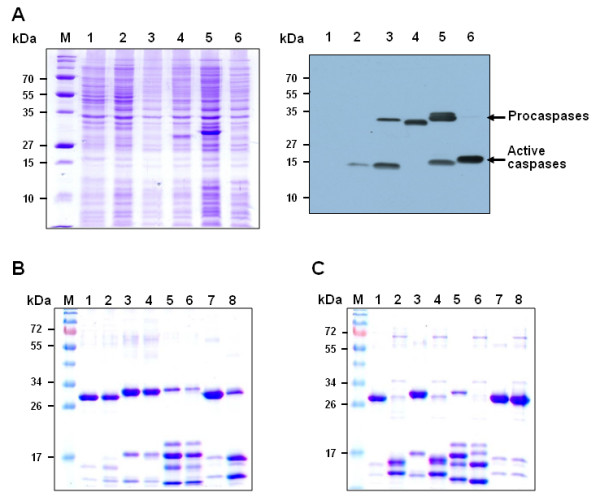
**SDS-PAGE analysis of engineered caspase-3 proteins**. A. Expression and Western blot analysis of engineered caspase-3 proteins. Cell lysates (20 μg) were prepared using an identical method, and loaded onto each well. M, molecular weight size marker; lane 1, uninduced *E. coli *lysate expressing wild-type caspase-3; lane 2, IPTG-induced *E. coli *lysate expressing wild-type caspase-3 (I); lane 3, IPTG-induced *E. coli *lysate expressing Δ28-caspase-3 (II); lane 4, IPTG-induced *E. coli *lysate expressing Δ28/175TS-caspase-3 (III); lane 5, IPTG-induced *E. coli *lysate expressing 28TS/175TS-caspase-3 (IV); lane 6, IPTG-induced *E. coli *lysate expressing 28TS/180TI-caspase-3 (V). The right and left images were obtained after coomassie blue staining and Western blot analysis, respectively. B. Treatment of caspase-3 precursors with wild-type caspase-3 (I). After each caspase-3 precursor was treated with wild-type caspase-3 (I) at RT for 18 h, 20 μl of each sample was heat-denatured and analyzed by SDS-PAGE. M, molecular weight size marker; lane 1, III; lane 2, III processed with I; lane 3, IV; lane 4, IV processed with I; lane 5, V; lane 6, V processed with I; lane 7, C163S caspase-3; lane 8, C163S caspase-3 treated with I. C. Activation of caspase-3 precursors by thrombin. Caspase-3 precursors were treated with thrombin at 4°C for 18 h, and 20 μl of each sample was heat-denatured and analyzed using PAGE. M, molecular weight size marker; lane 1, III; lane 2, III processed with thrombin (VI); lane 3, IV; lane 4, IV processed with thrombin (VII); lane 5, V; lane 6, V processed with thrombin (VIII); lane 7, C163S caspase-3; lane 8, C163S caspase-3 processed with thrombin.

Proteins were expressed in *E. coli *BL21 Rosetta (Novagen), and purified by cobalt affinity chromatography. Compared with wild-type, engineered proteins **III**, **IV **and **V **were highly expressed (Fig. 2A) and purified up to levels of 10–15 mg from 1 L *E. coli *culture. Interestingly, protein **V **displayed severe fragmentation during expression, despite the removal of known cleavage sites by site-directed mutagenesis. Since catalytically inactive C163S caspase-3 did not show a significant cleavage during expression, we suspected that self-catalysis by the precursor proteins is responsible for cleavage of proteins **V**. Hence, purified precursors were treated with catalytically active caspase-3 to establish whether auto-processing by precursor proteins was eliminated by mutation. While the C163S precursor protein was cleaved into two small fragments, no significant fragmentation was evident with the other proteins including protein **V**, implying that mutation of auto-cleavage sites blocks the auto-processing of caspase-3 precursors during expression (Fig. 2B). This finding also suggests that the cleavage of protein **V **in *E. coli *does not result from caspase activity, but that of another protease.

### Activation of engineered caspase-3 precursors

**Figure 3:**
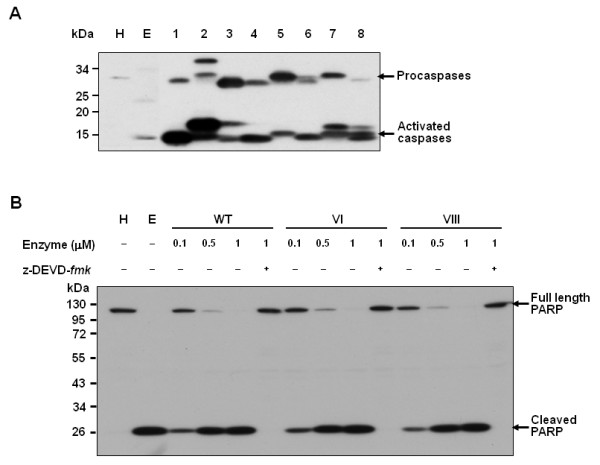
**Comparison of engineered caspases with endogenous caspase-3 and cleavage of PARP by engineered caspases**. A. Western blot analysis using anti-caspase-3 antibody. H, unstimulated HL-60 cell lysate; E, HL-60 cell lysate stimulated with etoposide; lane 1, purified wild-type caspase-3 (I); lane 2, purified II; lane 3, purified III; lane 4, VI; lane 5, purified IV; lane 6, VII; lane 7, purified V; lane 8, VIII. B. Western blot analysis of PARP cleavage by wild-type and engineered caspases (VI and VIII). HL-60 cell lysates were treated with each caspase in the presence and absence of caspase-3 inhibitor (*z*-DEVD-*fmk*) and Western blot analysis was carried out using an anti-PARP antibody which recognizes the full length (115 kDa) PARP and its cleavage product (24 kDa). H, unstimulated HL-60 cell lysate; E, HL-60 cell lysate stimulated with etoposide.

Purified proteins **III **and **IV**, together with the catalytically inactive C163S caspase-3 precursor, were treated with thrombin. As shown in Fig. 2C, ~30 kDa single polypeptides of **III **and **IV **were cleaved into two smaller peptides with molecular weights of 12 and 17 kDa (lanes 2 and 4), respectively. However, the C163S caspase-3 precursor did not display significant cleavage under similar conditions (lanes 7 and 8), indicating that fragmentation of **III **and **IV **is due to specific thrombin activity at the mutated sites. Notably, the cleavage sites of protein **V **during expression were distinct to those of thrombin. These specific sites remain to be identified. Since engineered caspase-3 precursors have His_6_-tag at their C-terminus, the thrombin can be easily removed by cobalt affinity chromatography after activation of caspase-3 (data not shown). Next, the size and activity of activated engineered caspases were compared with endogenous active caspase-3 using PAGE analysis. As shown in Fig. 3A, all engineered caspases activated by thrombin treatment were detected at similar position to endogenous active caspase-3. Furthermore, proteins **VI **and **VIII **cleaved PARP, a well-known substrate of caspase-3, at the similar level to wild-type caspase-3 (Fig. 3B).

### Kinetic analysis of caspase-3 precursors and activated proteins

The kinetic properties of caspase-3 precursors and their activated proteins were characterized using a commercially available synthetic substrate (Ac-DEVD-*p*NA). The engineered protein precursors **III **and **IV **containing LVPRGS exhibited negligible catalytic activity (no higher than 0.1% that of the wild-type enzyme). Thrombin digestion of proteins **III **and **IV **resulted in a drastic increase in catalytic activity (almost by two orders of magnitude), in agreement with a previous report [23] (Table 1). For example, *k*_cat_/*K*_*M *_values representing the substrate specificities of **IV **and **VII **were determined as 1.7 × 10^2 ^M^-1^*s*^-1 ^and 2.9 × 10^4 ^M^-1^*s*^-1^, respectively. The *k*_cat_/*K*_*M *_value of protein **V **containing an insertion of the LVPRGS motif between D180 and D181 was calculated as 6.7 × 10^4 ^M^-1^*s*^-1^, which was not significantly increased upon thrombin digestion. The data indicate that protein **V **is already activated during expression. This finding is supported by SDS-PAGE analysis results showing four smaller bands instead of the 30 kDa protein (Fig. 2A). Considering that Stennicke *et al*. attributed the marginal expression level of active caspase-3 in *E. coli *to the cytotoxicity of active caspase-3 [19], it was unexpected result. As the peptide sizes of protein **V **were further altered by digestion with thrombin but not wild-type caspase-3, we propose that cleavage during expression is attributed to the activity of another protease in *E. coli*. The present results are inconsistent with previous reports showing that uncleavable procaspase-3 mutants do not show this type of cleavage or meaningful activity [22]. It is envisaged that insertion of the LVPRGS peptide induces a structural change in procaspase-3 to some degree. Given that the catalytic cysteine shifts to an insignificant extent before and after the activation of caspase-7 [24], insertion of 6 amino acids into the loop containing catalytic Cys163 may be sufficient for its rearrangement to induce an active state, even without cleavage.

**Table 1:** Catalytic parameters of the caspase-3 mutants

ID	Caspase	*K*_M_(*μM*)	*k*_cat_(*s*^-1^)	*k*_cat_/*K*_M _(M^-1^*s*^-1^)
**I**	Wild type	14.16 ± 1.5	7.21 ± 0.93	(5.0 ± 0.1) × 10^5^
**II**	Δ28	16.38 ± 3.6	3.39 ± 0.58	(2.1 ± 0.1) × 10^5^
**III**	Δ28/175TS	93.20 ± 33.2	0.05 ± 0.02	(5.0 ± 0.0) × 10^2^
**IV**	28TS/175TS	93.49 ± 9.2	0.02 ± 0.00	(1.7 ± 0.0) × 10^2^
**V**	28TS/180TI	18.09 ± 1.3	1.26 ± 0.03	(6.7 ± 0.9) × 10^4^
**VI**	Δ28/175TS processed with thrombin	41.13 ± 1.9	1.86 ± 0.09	(4.5 ± 0.0) × 10^4^
**VII**	28TS/175TS processed with thrombin	27.81 ± 5.7	0.78 ± 0.06	(2.9 ± 0.4) × 10^4^
**VIII**	28TS/180TI processed with thrombin	21.39 ± 1.9	1.77 ± 0.13	(8.3 ± 0.1) × 10^4^

Significant changes in *k*_cat_, rather than *K*_*M *_values induced by mutation or activation resulted in similar substrate binding in all protein mutants, but altered catalytic activity. The *K*_*M *_and *k*_cat _values of protein **III **were 93.2 μM and 0.05 *s*^-1^, while those of **VI **were 41.3 μM and 1.86 *s*^-1^, respectively. The data correspond to 2.3 and 37.2-fold changes in substrate binding affinity and catalytic activity, respectively, upon activation. These findings imply that mutation or activation processes do not significantly alter the global shape of the active site, but affect the spatial arrangement of catalytic Cys163, in agreement with a previous report showing that activation of caspase-7 leads to rearrangement of a loop containing the catalytic cysteine [24].

## Conclusion

In this study, we demonstrate a novel strategy for a large-scale preparation of active caspase-3 in *E. coli*. Thrombin-activatable caspase-3 precursors were designed to suppress autoprocessing, and their activation regulated by thrombin. The designed precursors were highly expressed in *E. coli*, easily purified, and activated by thrombin digestion, yielding 10–15 mg of active caspase-3 from 1 L of *E. coli *culture. Following thrombin activation, catalytic activity was increased about 100-fold, analogous to previous findings on the wild-type precursor and mature caspase-3 protein. This research represents the first example to highly express a full-length caspase-3 as a soluble protein in *E. coli*, facilitating a large-scale preparation of active caspase-3. This system may be effectively applied to prepare other caspases on a large scale.

## Methods

### Materials

Primer synthesis and DNA sequencing were performed by Bioneer (Daejeon, Korea) and Genotech (Daejeon, Korea), respectively. The primers used for plasmid construction are listed in Table 2. The caspase substrate, Ac-DEVD-*p*NA, was purchased from Anaspec, and thrombin from Roche. Protein expression and cleavage were monitored by 15% SDS-PAGE analysis. Caspase activity was measured by monitoring the absorption changes at 405 nm, based on the release of *p*-nitroanilide from substrate hydrolysis, on a DU 800 UV-VIS spectrophotometer (Beckman Coulter). Amino acid sequences of the engineered proteins were numbered based on wild-type procaspase-3.

**Table 2:** PCR primers used for plasmid construction

Primer	5' → 3'
**P1**	GGGAATTCCATATGGAGAACACTGAAAACTCAG
**P2**	CCGCTCGAGGTGATAAAAATAGAGTTCTTTTGT
**P3**	GGGAATTCCATATGTCTGGAATATCCCTGGACAACAGT
**P4**	ATCAAC*GCTGCCGCGCGGCACCAG*GCCACAGTCCAGTTCTGTACCACG
**P5**	TGTGGC*CTGGTGCCGCGCGGCAGC*GTTGATGATGACATGGCGTGTCAT
**P6**	ACTGTTGTCCAGGGATAT*GCTGCCGCGCGGCACCAG*GCTTCCATGTATGATCTT
**P7**	GACTGTGGCATTGA*GAC*AGCGAGTGGTGTTGATGAT
**P8**	CAGATCATCAACACCACTCGCT*GTC*TCAATGCCACA
**P9**	ACAGACAGTGGTGTTGATGAT*CTGGTGCCGCGCGGCAGC*GACATGGCGTGTCATAAA
**P10**	ACAGACAGTGGTGTTGATGAT*CTGGTGCCGCGCGGCAGC*GACATGGCGTGTCATAAA

* *Nde*I restriction sites in P1 and P3, and *Xho*I site in P2 are underlined. Thrombin recognition sites in P4, P5, P6, P9 and P10 and Ala mutations in P7 and P8 are italicized.

### Generation of caspase-3-overexpressing constructs

Wild-type caspase-3 gene was amplified using the forward primer, 5'-GGGAATTC**CATATG**GAGAACACTGAAAACTCAG-3' (**P1**) containing an *Nde*I site (marked in bold type letters), and reverse primer, 5'-CCG**CTCGAG**GTGATAAAAATAGAGTTCTTTTGT-3' (**P2**), with a *Xho*I site (underlined), and inserted into the corresponding sites of the pET21a plasmid (Novagen). The resulting construct was designated plasmid **I**. A Δ28 caspase-3 deletion mutant devoid of the N-terminal 28 amino acids of wild-type caspase-3 (**I**) was prepared using the forward primer, 5'-GGGAATTC**CATATG**TCTGGAATATCCCTGGACAACAGT-3' (**P3**) with an *Nde*I site (marked in bold type letters), and reverse primer, **P2**. The resulting DNA was inserted into the pET21a expression vector, and denoted plasmid **II**. For preparation of Δ28/175TS caspase-3, the 175TS mutant of plasmid **I **was initially prepared using the megaprimer PCR method with slight modifications [25]. N-terminal megaprimer DNA encoding amino acids 1~177, which is substituted at positions 172~177 (IETDSG) with LVPRGS, was prepared using the forward primer, **P1**, and reverse primer, 5'-ATCAAC*GCTGCCGCGCGGCACCAG*GCCACAGTCCAGTTCTGTACCACG-3' (**P4**). C-terminal megaprimer DNA substituted at positions 172–177 (IETDSG) with LVPRGS was prepared using the forward primer, 5'-TGTGGC*CTGGTGCCGCGCGGCAGC*GTTGATGATGACATGGCGTGTCAT-3', (**P5**) and reverse primer, **P2**. The thrombin recognition sites in **P4 **and **P5 **are italicized. Each megaprimer was extended by PCR, using the other as a template. The resulting DNA was inserted into pET21a using the *Nde*I and *Xho*I restriction sites, and designated plasmid **IX**. The Δ28/175TS caspase-3 mutant of plasmid **IX **was prepared using forward **P3 **and reverse **P2 **primers. The amplified product was inserted into the *Nde*I and *Xho*I sites of pET21a, and the construct denoted plasmid **III**. For generating the 28TS/175TS mutant caspase-3 construct in plasmid **IX**, the megaprimer with substitutions at positions 24–30 (LVPRGS in place of ESMDSG) was prepared using the forward primer, **P1**, and reverse primer, 5'-ACTGTTGTCCAGGGATAT*GCTGCCGCGCGGCACCAG*GCTTCCATGTATGATCTT-3' (**P6**). The thrombin recognition site in **P6 **is italicized. The resulting megaprimer, in combination with **P2**, was applied for the next PCR reaction to prepare 28TS/175TS caspase-3 DNA, which was cloned into pET21a, and designated plasmid **IV**. The 28TS/D175A/180TI caspase-3 construct was generated in plasmid **I **using a combination of megaprimer PCR and the QuikChange site-directed mutagenesis kit (Stratagene). To prepare the 28TS caspase-3 construct in plasmid **I**, a corresponding megaprimer produced using the P1 forward and P6 reverse primers was employed together with **P2**. The resulting construct was designated plasmid **X**. A 28TS/D175A caspase-3 construct in plasmid **X **was prepared with the QuikChange kit using the forward primer, 5'-GACTGTGGCATTGA*GAC*AGCGAGTGGTGTTGATGAT-3' (**P7**), and reverse primer, 5'-CAGATCATCAACACCACTCGCT*GTC*TCAATGCCACA-3' (**P8**), and designated plasmid **XI**. The Ala mutation sites in **P7 **and **P8 **are italicized. The thrombin recognition site was successfully inserted into the plasmid **XI **background with the megaprimer method. N- and C-terminal megaprimers were prepared using the forward primer, **P1**, and reverse primer, 5'-TATTTTATGACACGCCATGTC*GCTGCCGCGCGGCACCAG*ATCATCAACACCACT-3' (**P9**), as well as the forward primer, 5'-ACAGACAGTGGTGTTGATGAT*CTGGTGCCGCGCGGCAGC*GACATGGCGTGTCATAAA-3' (**P10**) and reverse primer, **P2**, respectively. The thrombin recognition sites are italicized. Each prepared megaprimer was extended by PCR, using the other as a template, and the resulting product inserted into the *Nde*I and *Xho*I sites of pET21a (designated plasmid **V)**.

### Preparation of recombinant caspase-3 precursors

*E. coli *BL21 Rosetta cells (Novagen) containing the specified expression plasmids were grown at 37°C in LB medium until an *A*_600 _of 0.6–0.8. Engineered caspase-3 precursors were expressed by adding 1 mM IPTG at 18°C for 18 h. Cells were harvested, washed with buffer A (50 mM Tris pH 7.5, 250 mM NaCl, 5% glycerol, and 0.05% β-mercaptoethanol), and lysed by ultrasonication. After centrifugation (29,820 *g *for 30 min), the supernatant was incubated with a cobalt affinity resin (TALON^®^, Clontech) on a rocker at 4°C for 1 h, and washed with buffer A containing 10 mM imidazole. Proteins were eluted from the metal affinity resin with buffer A supplemented with 100 mM imidazole. Following dialysis against 20 mM Tris, pH 7.5, 250 mM NaCl, 5% glycerol, and 1 mM dithiothreitol, caspase-3 precursors were concentrated to 2 mg/ml and stored at -80°C.

### Activation of caspase-3 precursors by thrombin

Caspase-3 precursors were activated by digestion with bovine thrombin. Briefly, 100 μg of each precursor protein was incubated with 1 NIH unit of thrombin in 50 mM Tris, pH 7.5, 250 mM NaCl, 5% glycerol, 0.05% β-mercaptoethanol, 3 mM CaCl_2 _at 4°C. Digestion was performed for 18 h, and monitored by 15% SDS-PAGE.

### Treatment of caspase-3 precursors with wild-type caspase-3

Caspase-3 precursors were treated with wild-type caspase-3 to test autocleavage during activation. Briefly, 100 μg of each precursor protein was incubated with 1 μg of wild-type caspase-3 in 50 mM Tris, pH 7.5, 250 mM NaCl, 5% glycerol, 0.05% β-mercaptoethanol, and 5 mM DTT at RT. Cleavage was performed for 18 h, and monitored by 15% SDS-PAGE.

### Determination of caspase-3 activity and kinetic constants

The activity of each caspase-3 protein was measured using the colorimetric substrate, Ac-DEVD-*p*NA. Briefly, caspase-3 was activated in reaction buffer (50 mM HEPES, pH 7.4, 50 mM KCl, 2 mM MgCl_2_, 1 mM EDTA, and 5 mM dithiothreitol) for 18 h before use. The appropriate amount of activated caspase-3 (final concentrations of 10–80 nM) was added to the substrate solution at a series of final concentrations (0, 12.5, 25, 50, 100 and 200 μM). The *p*-nitroanilide released by the caspase reaction was monitored at 405 nm using a DU 800 UV-VIS Spectrophotometer (Beckman Coulter). *K*_m _and *V*_max _values were obtained using Hyper32 version 1.0.0 , and *k*_cat _values calculated from *V*_max _and the used enzyme concentration.

### Cleavage of endogenous PARP by engineered caspases

Human promyelocytic leukemia HL60 cells were cultured in RPMI. If necessary, apoptosis was induced by treatment of the cells with 100 μM etoposide for 12 h. Cell lysates (60 μg) were incubated with engineered caspases at 37°C for 2 h. Then, the cleavage of endogenous PARP was determined by immunoblot analysis with an anti-PARP antibody. For negative control, cell lysates treated together with engineered caspases and z-DEVD-*fmk *were used [26,27].

## Authors' contributions

HJK, SGP and KHB participated in the experimental design, carried out the molecular genetic and biochemical experiments, participated in data interpretation and helped draft the manuscript. YML, YJJ, KP, MJ and MK conceived the study. SJC directly supervised the project, participated in its experimental design and data interpretation and was responsible for writing the manuscript. All authors have read and approved the manuscript.
